# *BRCA1* Norway: comparison of classification for *BRCA1* germline variants detected in families with suspected hereditary breast and ovarian cancer between different laboratories

**DOI:** 10.1007/s10689-021-00286-6

**Published:** 2022-01-04

**Authors:** Henrikke N. Hovland, Rafal Al-Adhami, Sarah Louise Ariansen, Marijke Van Ghelue, Wenche Sjursen, Sigrid Lima, Marte Bolstad, Amund H. Berger, Hildegunn Høberg-Vetti, Per Knappskog, Bjørn Ivar Haukanes, Ingvild Aukrust, Elisabet Ognedal

**Affiliations:** 1 Western Norway Familial Cancer Center, Haukeland University Hospital, Bergen, Norway; 2 Department of Medical Genetics, Haukeland University Hospital, Bergen, Norway; 3 Department of Clinical Science, University of Bergen, Bergen, Norway; 4 Department of Medical Genetics, Oslo University Hospital, Oslo, Norway; 5 Department of Medical Genetics, University Hospital of North Norway, Tromsø, Norway; 6 Department of Medical Genetics, St. Olavs University Hospital, Trondheim, Norway

**Keywords:** Breast and ovarian cancer, *BRCA1*, Variant classification, Variants of uncertain significance

## Abstract

Pathogenic germline variants in *Breast cancer susceptibility gene 1* (*BRCA1*) predispose carriers to hereditary breast and ovarian cancer (HBOC). Through genetic testing of patients with suspected HBOC an increasing number of novel *BRCA1* variants are discovered. This creates a growing need to determine the clinical significance of these variants through correct classification (class 1–5) according to established guidelines. Here we present a joint collection of all *BRCA1* variants of class 2–5 detected in the four diagnostic genetic laboratories in Norway. The overall objective of the study was to generate an overview of all *BRCA1* variants in Norway and unveil potential discrepancies in variant interpretation between the hospitals, serving as a quality control at the national level. For a subset of variants, we also assessed the change in classification over a ten-year period with increasing information available. In total, 463 unique *BRCA1* variants were detected. Of the 126 variants found in more than one hospital, 70% were interpreted identically, while 30% were not. The differences in interpretation were mainly by one class (class 2/3 or 4/5), except for one larger discrepancy (class 3/5) which could affect the clinical management of patients. After a series of digital meetings between the participating laboratories to disclose the cause of disagreement for all conflicting variants, the discrepancy rate was reduced to 10%. This illustrates that variant interpretation needs to be updated regularly, and that data sharing and improved national inter-laboratory collaboration greatly improves the variant classification and hence increases the accuracy of cancer risk assessment.

**Supplementary Information:**

The online version contains supplementary material available at 10.1007/s10689-021-00286-6.

## Introduction

While most cancer cases are sporadic, 5–10% are hereditary and caused by disease-causing germline variants in cancer susceptibility genes. Hereditary breast and ovarian cancer (HBOC) can be caused by alterations in several genes, among which the tumour suppressors *Breast cancer susceptibility gene 1* and 2 (*BRCA1* and *BRCA2*) are the most prevalent and studied. Carriers of (likely) pathogenic germline variants of *BRCA1*, which is the focus of this study, have a lifetime risk of 56–75% for breast cancer and 36–51% for ovarian cancer [1].

In recent years, technological development and reduced costs have led to rapid growth in the use of genetic testing of patients with suspected HBOC. An increasing number of novel *BRCA1* variants are thus being discovered, and to date more than 11 000 *BRCA1* variants are registered in ClinVar [2]. Accurate assessment of the clinical relevance of a given *BRCA1* variant is crucial for risk assessment, genetic counselling, and clinical management including cancer prevention in both the patient and healthy relatives with the same hereditary predisposition. A joint consensus of standards and guidelines for the interpretation of genetic variants in general has been made by ACMG-AMP (The American College of Medical Genetics and Genomics and the Association for Molecular Pathology) [3]. The pathogenicity of a variant is interpreted according to a five-tier score system with the following designations: benign (class 1), likely benign (class 2), uncertain significance (class 3), likely pathogenic (class 4), and pathogenic (class 5) [4]. In addition, the expert consortium ENIGMA (Evidence-based Network for the Interpretation of Germline Mutant Alleles) has developed classification criteria specific for *BRCA1* and *BRCA2* [5].

While *BRCA1* variants classified as either likely benign or benign are not associated with increased risk of cancer, variants classified as likely pathogenic or pathogenic increase the risk of cancer by impairing protein structure or function. Carriers of such variants are offered regular surveillance and prophylactic surgery according to national guidelines [6–8]. However, for a large number of *BRCA1* variants the knowledge is either very limited or conflicting, and accordingly these are classified as variants of uncertain significance (VUS). The expanding use of genetic testing increases the number of new and rare VUSs identified. Hence, even though *BRCA1* is a well-characterized gene, interpretation of variants in this gene is still a challenge for the individual clinical laboratories.

Discrepancies in the interpretation of the same gene variants at different laboratories have previously been observed in several countries, including Canada and USA [9–12]. The consequences can be tragic. Recently, an example of misinterpretation was unveiled in a hospital in Norway, where twenty-one female carriers had their breast and/or ovaries removed by prophylactic surgery without sufficient evidence that their variant, *BRCA2* (NM_000059.3) c.68–7 T > A, was pathogenic [13, 14]. The other Norwegian hospitals did not classify this *BRCA2* variant as pathogenic, but this was unknown at the time, as there is no general practice for data sharing or a common national variant database. There are several serious consequences of a misclassified variant including unnecessary interventions in patients and misallocation of resources for the society. Furthermore, family members harbouring the same genetic variant may receive different medical advice if they live in different parts of the country. This may lead to increasing uncertainty and anxiety among carriers of such variants.

In this study, based on inter-laboratory collaboration, we aim to give an overview of all class 2–5 *BRCA1* variants identified at the four diagnostic genetic laboratories in Norway. Furthermore, we compare the corresponding classifications at the different hospitals to explore the national consistency of *BRCA1* variant interpretation. In addition, for a subset of variants, we aim to assess the change in classification over time with increasing information available. Ideally, the collaboration will give an increased consensus regarding *BRCA1* variant classification and create a forum for future discussions.

## Materials and methods

*BRCA1* variants were collected from the four diagnostic genetic laboratories in Norway; Haukeland University Hospital in Bergen (HUH, 177 variants), Oslo University Hospital (OUH, 303 variants), the University Hospital of North Norway in Tromsø (UNN, 88 variants) and St. Olav’s University Hospital in Trondheim (TUH, 84 variants). All *BRCA1* variants had been detected by genetic testing of patients or healthy family members of patients with suspected HBOC from late 1990s to July 2019. Samples were mainly analysed by Sanger sequencing and/or NGS (Illumina custom made gene panel). Each variant was classified at the hospitals according to local protocols based on the ACMG-AMP guidelines or equivalent procedures. Nomenclature was assigned according to the Human Genome Variation Society (HGVS), and the reference sequence NM_007294.3 was used [15]. Variants reported as benign (class 1) and copy number variants identified by multiplex ligation-dependent probe amplification (MLPA) were not included in the dataset.

For VUSs observed in only one hospital and with a classification report older than three years in 2019 (*n* = 45), a reassessment of the variants was performed by HUH. For variants detected in more than one hospital, classifications were compared. For all variants with conflicting classifications, the corresponding laboratories were asked to reassess the variant. Following this reclassification, a series of digital meetings between all laboratories were arranged to disclose the cause of disagreement and to try to reach consensus. At least one laboratory geneticist from each hospital participated in these discussions.

Finally, for a subset of variants (variants found at HUH in the period from 2007 to 2017 (*n* = 115)) the classifications at HUH and ClinVar over time were compared. A heat map of these classifications was generated using R (v. 4.0.2) and the package ggplot2 [16]. Data was cleaned and managed with tidy data principles using the tidyverse collection of packages (v 1.3.1) [17]. The colour scale used for variant classification was generated using the RColorBrewer package (v. 1.1–2). Multiple heat maps and a bar plot were combined using the package cowplot (v. 1.1.1).

The program Alamut Visual (Version 2.13) was used as a tool during reassessment of variants [18]. The variant allele frequencies were retrieved from GnomAD v2.1.1 [19].

## Results

**Fig. 1:**
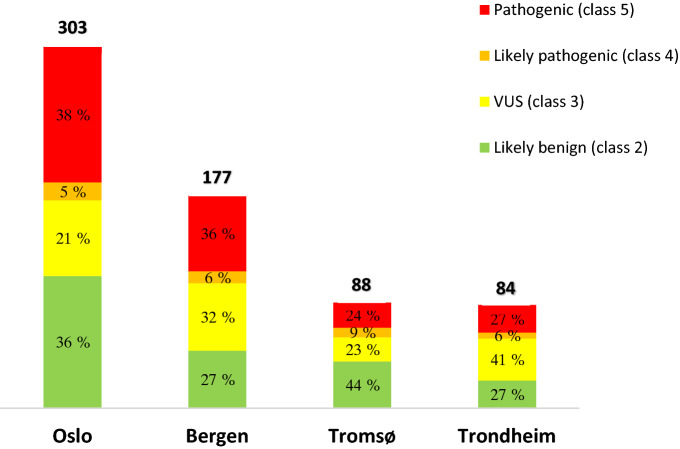
Number of *BRCA1* variants submitted from the participating hospitals and the local distribution of variants within each class. In total 652 variants were submitted from the four laboratories

In total, 652 *BRCA1* variants were submitted from the four hospitals. The number of variants from each hospital, in addition to the distribution of variants within each class, is shown in Fig. 1. After removal of overlapping variants, 463 *BRCA1* variants were shown to be unique (Supplementary table 1). Of the 463 unique variants, 126 variants (27%) were detected in more than one hospital; 76 (16%), 37 (8%) and 13 (3%) variants were detected at two, three and four hospitals, respectively (Table 1). The remaining 337 (73%) *BRCA1* variants were observed in one hospital only.

**Table 1:** Number of *BRCA1* variants detected in more than one hospital

Number of hospitals	Number of variants	Percentage
1	337	73
2	76	16
3	37	8
4	13	3
Total	463	100

After removal of overlapping variants, 463 variants were shown to be unique. 126 variants were detected in more than one hospital, while the remaining 337 variants were observed in one hospital only

**Fig. 2:**
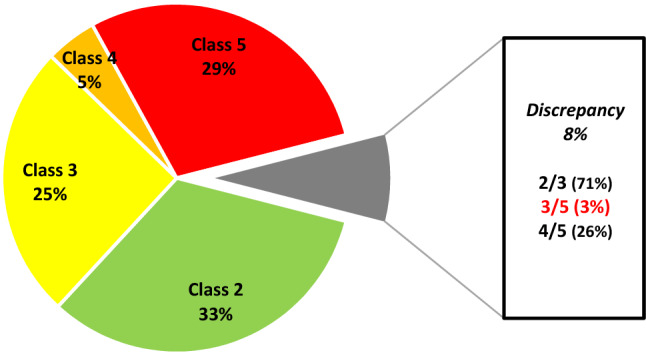
Distribution of *BRCA1* variants within each pathogenicity class

**Fig. 3:**
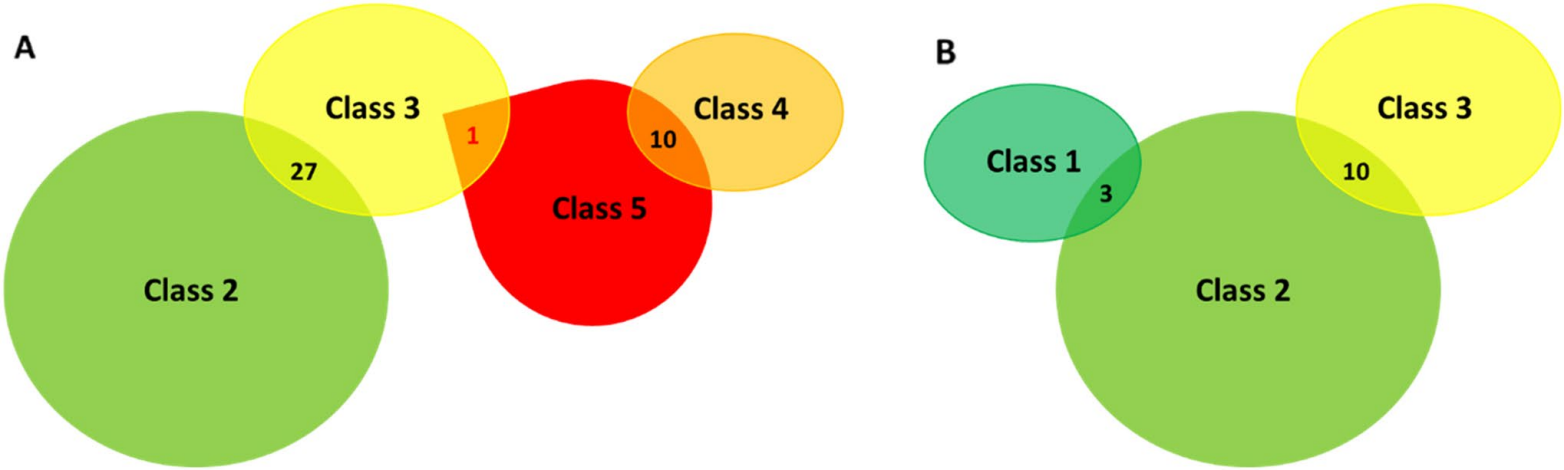
Distribution of *BRCA1* variants with conflicting interpretations. **A** For 38 of the variants detected in more than one hospital there were discrepancies in interpretations between the hospitals. The majority of discrepancies (37/38) were one class apart. Only one variant was found to have a discrepancy extending two classes. **B** After a series of collaborative meetings between the different hospitals to discuss the causes of disagreement, the number of conflicting classifications were reduced to 14. All discrepancies were one class apart, mainly between class 2 and 3

For the 126 variants detected in more than one hospital, the corresponding classifications were compared. For 30% (38/126) of these variants, there were discrepancies in interpretations between the hospitals (Table 2). The differences in interpretation were mainly by one pathogenicity class (class 2/3 or 4/5) as shown in Figs. 2 and 3A. Alarmingly, one class 3/5 discrepancy was detected for the variant *BRCA1* c.457_458ins21. This variant was observed in three hospitals; one of the hospitals reported the variant as class 3 (variant of uncertain significance), while the two other hospitals reported it as class 5 (pathogenic). In the class 5 reports, the variant was described as an insertion of 21 nucleotides leading to a premature stop codon (ATTAGCA*G*GAAACCAGTCTCA). This did not correspond with the class 3 report, where the inserted nucleotide sequence was different, and did not contain a stop codon (ATTA*C*CA*A*GAAACCAGTCTCA). Thorough investigations of the raw data revealed that the discrepancy was caused by a misread of the inserted sequence due to software weakness (Sequence Pilot, JSI medical systems), and that a stop codon was indeed present in the insertion. The mistake was corrected and all hospitals now classify *BRCA1* c.457_458ins21 as pathogenic (class 5).

**Table 2:** *BRCA1* variants with conflicting classification between different Norwegian hospitals and resulting reclassification after collaboration

Variant	Oslo (OUH)		Bergen (HUH)		Tromsø (UNN)		Trondheim (TUH)		Reclassified	
	Class	Date	Class	Date	Class	Date	Class	Date	Class	Date
c.19C > T	3	2015	2	2015	3	2018			3	2021
c.140G > T	4	2014	5	2008					4	2021
c.301 + 7G > A					3	2013	2	2015	2^∆○^, 1^□^	2021
c.441G > C	3	2017	3	2013	2	2010			3	2021
c.441 + 21C > T			2	2018			3	2018	3	2021
c.457_458ins21	5	2014	3	2016			5	2016	5	2019
c.547 + 14del			3	2011	2				2^∆^,1^□^	2021
c.557C > A			3	2014			2	2015	2	2021
c.670 + 16G > A			3	2018	2	2010	2	2016	2	2021
c.736 T > G			3	2014	2	2010	2	2018	2	2021
c.889A > G	2	2019	3	2016					2*,3^∆^	2021
c.1287del	5	2008	5	2015	4	2014			5	2021
c.1508A > G	2	2017					3	2017	2*^○^,3^∆^	2021
c.1534C > T	2	2018	2	2018	3	2015	3	2018	2	2021
c.1568 T > G	2	2018			3	2017	3	2017	2*^○^,3^∆□^	2021
c.1687C > T	5	2018	4	2013	5	2018			5	2021
c.1772 T > C	2	2019	3	2011					2	2021
c.1879G > A	2	2018	3	2019	3	2016			2*,3^∆□^	2021
c.2131A > C	2	2019	3	2019					2*,3^∆^	2021
c.2183G > A	2	2018			3	2016			2*,3^∆□^	2021
c.2315 T > C	3	2015					2	2019	1*,2^∆○^	2021
c.2773A > G	2	2017	3	2014					2*,3^∆^	2021
c.2798G > A	2	2018	3	2016					2*,3^∆^	2021
c.3041 T > A	3	2012	2	2016					3	2021
c.3228_3229del	5	2018	5	2019	4	2012	5	2018	5	2021
c.3319G > T	5	2018			4	2010			5	2021
c.3454G > A	2	2018	3	2018					2	2021
c.3640G > A	3	2010	2	2019					2	2021
c.3659A > T	2	2018			3	2012	3	2018	2*,3^∆□○^	2021
c.4096 + 3A > G	3	2019	2	2017			3	2017	3	2021
c.4300del	5		5	2015	4	2016			5	2021
c.4315C > T	2	2019	3	2019	3	2018	3	2018	2*,3^∆□○^	2021
c.4484G > A	5	2015			5	2015	4	2015	5	2021
c.5047G > T	5	2018	5	2008	4	2014			5	2021
c.5096G > A	4	2018	4	2013	5	2019	5	2017	5	2021
c.5213G > A	5		4	2010					5	2021
c.5348 T > C	2	2017	3	2018					2	2021
c.5477A > T	2	2017			3	2016	3	2017	2	2021

For *BRCA1* variants with conflicting classifications after reassessment, the following symbols indicate the corresponding laboratories: * = OUH (Oslo University Hospital), ^∆^ = HUH (Haukeland University Hospital in Bergen), ^□^ = UNN (University Hospital of North Norway in Tromsø), ^○^ = TUH (St. Olav’s University Hospital in Trondheim)

Comparison of the *BRCA1* variants in Supplementary table 1 with previously published *BRCA1* variants found in certain regions of Norway [20–22] revealed that the two variants *BRCA1* c.5123C > T (VUS) and c.4883 T > C (likely benign) have previously been incorrectly classified as pathogenic in a recent publication [20]. The authors were informed on the discovery, and the mistakes were later corrected [23]. According to the authors, the mistakes were caused by problems related to formatting of a table in the article, and the incorrect classifications had not been utilized in the clinic.

In order to ensure updated classifications, VUSs with only one interpretation report older than three years (*n* = 45) were reassessed. In total, eleven variants were reclassified to likely benign variants, while one variant was reclassified as a benign variant. The remaining 33 variants were still assessed as VUSs.

For the 38 variants with conflicting classifications between hospitals, each laboratory was asked to reassess the variants, resulting in a reduction of the rate of discrepancies from 30% (38/126) to 14% (18/126). All laboratories then participated in a series of digital meetings discussing the causes of disagreement, further reducing the discrepancy rate to 10% (13/126) (Fig. 3B).Thus, after reassessment of the variants, 66% (25/38) of the original conflicting interpretations eventually reached consensus.

**Fig. 4:**
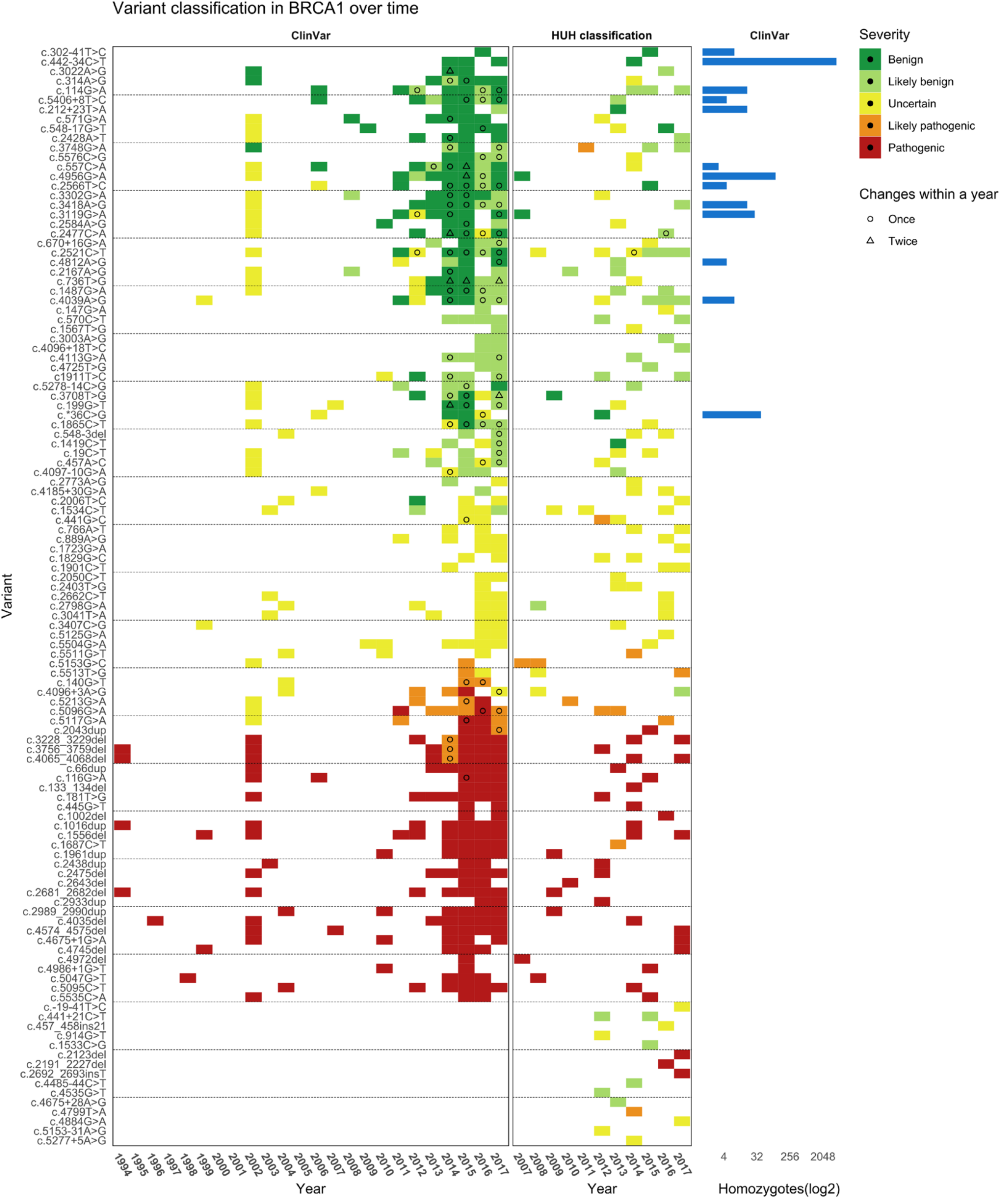
Heat map: Schematic heat map presentation of changes in classification over time for variants detected at Haukeland University hospital in the period from 2007 to 2017. The figure presents classifications performed locally at the hospital compared to classifications reported to the open access database ClinVar in the same timeframe

For a sub-cohort of the variants (detected at HUH in the period from 2007 to 2017) a schematic presentation of their classification over time at the hospital as well as in ClinVar was made. The heat map shows that among the variants that changed classification over time, the majority were VUSs reclassified to likely benign both at HUH and ClinVar (Fig. 4). The following were observed; (1) nine variants from HUH and 22 variants from ClinVar were downgraded, (2) three variants from HUH and eight variants from ClinVar were upgraded, and (3) no variants from HUH and 16 variants from ClinVar were both upgraded and downgraded. Fifteen of the variants from HUH were not reported in ClinVar. The concordance in classifications between HUH and ClinVar was relatively high.

## Discussion

Even though *BRCA1* sequencing of HBOC patients has been performed in Norway since late 1990s, previous studies characterizing *BRCA1* variants in Norway have included only specific regions of the country [20–22, 24]. This study is the first to include *BRCA1* variant data from all four medical genetic departments in Norway, and gives a complete overview of the Norwegian *BRCA1* variant spectrum. Comparison of variant classification between the different hospitals revealed several discrepancies and clearly illustrates the pivotal role of sharing variant interpretation data. Furthermore, the change in variant classification over time for a subset of the data demonstrates the importance of updating variant classifications regularly.

Due to the complexity of variant interpretation, some discrepancies among hospitals are expected [9], and the discrepancy rate of 30% for Norwegian *BRCA1* variants found in this study is within the range of previous findings in similar studies [25]. A study from Canada that investigated variants uploaded to a national database by eleven participating diagnostic laboratories found that 38.9% (350/900) of the *BRCA1* and *BRCA2* variants classified by two or more laboratories had conflicting interpretations when using a five-tier classification model. After reassessment of the variants, 21.4% (75/350) of the conflicting interpretations reached consensus. The laboratories reported that the main reasons for reclassifying a variant was availability of new evidence (52.7%), and the use of revised classification criteria (28.4%) [11]. Several other studies performed on gene variants in general have found similar discrepancy rates [9, 10]. In addition, analysis of all gene variants reported to ClinVar has shown that 17% (2229/12895) of the variants submitted by more than one laboratory were interpreted differently [26]. Currently, 4% (374/8829) of *BRCA1* variants reported in ClinVar are registered with conflicting interpretations [2]. Some studies report discrepancy rates much lower than the examples described above [27, 28]. However, in contrast to our study, in these studies a five-tier classification system was not used, but rather a two-tier system reporting discrepancies only between variants described as non-actionable (class 1–3) and clinically actionable (class 4–5). These results are therefore still in concordance with the findings in our study, as we only found one discrepancy that would affect the management of patients. Overall, our results indicate that the *BRCA1* variant classification in Norwegian hospitals is relatively consistent.

Unveiling potential conflicting interpretations that may impact the management of patients is of high value. Of particular interest, this study revealed one classification deviation (class 3/5) for the variant *BRCA1* c.457_458ins21. There is a major difference in the clinical management of patients harbouring a VUS and a pathogenic *BRCA1* variant, and misclassification of this pathogenic variant as a VUS has serious consequences by depriving the affected family of appropriate treatment. Healthy carriers of pathogenic variants are offered surveillance and risk reducing surgery to prevent cancer [29–35], and accurate assessment of a genetic variant is crucial to ensure that carriers receive satisfactory genetic counselling regarding these options. Patients with *BRCA1* deficient cancers are also candidates for treatment with Poly (ADP-ribose) polymerase (PARP)-inhibitors, thus *BRCA1* variant interpretation status is extremely important for treatment decisions [36–41]. Accordingly, identification of a pathogenic *BRCA1* variant in the family affects both the patient and healthy family members who might have inherited the same variant. After discovering the misclassification, all family members were re-advised for further genetic testing and correct clinical management was offered. Luckily, no new cancer cases had occurred in the family during the period of misclassification (2016–2019).

The majority of the identified classification discrepancies did not affect the clinical management of patients. There were 27 variants with conflicting interpretation between class 2 (likely benign) and class 3 (VUS), and ten variants with conflicting interpretations between class 4 (likely pathogenic) and class 5 (pathogenic). As variants of both class 4 and 5 are clinically actionable, such discrepancies are of lower clinical relevance. If a likely benign variant is detected in a patient, it is assumed that this is not the explanation for the cancer in the family (but it does not rule out other hereditary causes). If a VUS is detected, it is not possible to determine if this is the cause of the cancer and the classification report will be inconclusive. A VUS is thus not clinically actionable [4], but it might still produce significant anxiety among the carriers if reported back to the patient. Such findings will often need further analysis of the variant like functional analysis, and reassessment of the pathogenicity when new knowledge is unveiled.

All 38 variants with conflicting classifications were discussed between the participating hospitals, aiming to disclose the cause of disagreements and increase the national consensus regarding *BRCA1* variant classification in Norway. The main reasons for conflicting classifications were found to be differences in how strictly the different laboratories followed the ACMG-AMP classification guidelines, in addition to different understandings of some of the guidelines. The BP1 evidence (missense variant in a gene for which primarily truncating variants are known to cause disease) was one of the most debated criteria. As only two supporting benign evidence are enough to classify a variant as likely benign according to ACMG-AMP, use of this evidence would more easily lead to classification of *BRCA1* missense variants outside the RING and BRCT domains as likely benign. Since there is only limited knowledge about the regions located outside these protein domains, it was debated whether or not this criteria should be used as supportive benign evidence. Several publications have suggested that most *BRCA1* missense substitutions located outside of critical domains could be classified as likely benign, arguing that pathogenic missense variants are infrequent in these regions. This is supported by the fact that the ClinVar dataset contains hardly any (likely) pathogenic *BRCA1* missense variants located outside the critical domains. It was however debated that this does not necessarily mean that such variants do not exist. During the folding of proteins, amino acid residues originally located outside well established domains in the primary structure can come in contact with important structural and functional elements in the three dimensional structure of the native folded protein. Thus, it is reasonable to believe that the consequence of introducing a missense variant involving an amino acid with major differences in the size, polarity and physiochemical properties compared to the original residue could be fatal, also for residues located outside well established domains. Since the structural knowledge of BRCA1 is sparse and there is only limited knowledge about the regions located outside these protein domains, functional studies similar to the saturation editing data for the BRCT and RING domains are needed to further address this issue. After the discussions, an agreement was made to use BP1 with caution, and always to compile with data on amino acid conservation as well as comparison of the physiochemical properties of the original and new amino acid residue. There were also differences in the use of the ACMG-AMP BS1 evidence (allele frequency is greater than expected for disorder) as some of the laboratories use different cut-off values regarding allele frequencies to decide the strength of the BS1 evidence. Some laboratories use new and updated guidelines like CanVig-UK [42] in addition to the ACMG-AMP guidelines. The degree of emphasis on classifications performed by the expert consortium ENIGMA were also the reason for some of the discrepancies. If ENIGMA had classified a variant as benign (class 1), some of the laboratories weighted this stronger than any of the ACMG-AMP guidelines. Other reasons for inter-lab discrepancies were in-house information regarding family history and findings of additional pathogenic variants in combination with the variant of interest.

Both the criteria for eligibility of having a genetic test and the criteria used to classify a variant have changed during the years included in the study. The ACMG-AMP guidelines for the interpretation of sequence variants were published in 2015. The ENIGMA criteria for classifying *BRCA1* variants were first published in 2009, and lastly updated in 2017. In addition, even though laboratories use standardized methods when interpreting variants, the available information is often sparse or sometimes even conflicting. Noteworthy, the resulting classification of a variant is dependent on the information available in the local laboratory at the time of interpretation. Thus, a given classification is most correct at the specific time of interpretation based on available information, but is outdated and should ideally be reassessed when new information is available. Such discrepancies can be solved by data sharing between the hospitals and regular reassessment of variants, but in Norway a variant is often only reassessed if it is identified in a new individual.

After observing that 12 of the 45 VUSs with old classification reports could be reclassified to likely benign after a new assessment, we wanted to further investigate how the *BRCA1* classification had potentially changed over time in general. Thus, the classification history for a subset of *BRCA1* variants reported between 2007 and 2017 at HUH and in ClinVar when available was generated. Both at HUH and in ClinVar the classification of several variants had changed over time following the rapid increase of available information. The majority of reclassified *BRCA1* variants were downgraded from VUS to (likely) benign variants in concordance with other studies reporting reclassification of *BRCA1* variants [43–49]. Most likely this is due to open-access databases like ClinVar (made available in 2012), and gradually increasing knowledge about variant frequencies in the general population made available in GnomAD in 2017 [19] and its precursor ExAC in 2014. Data on allele frequencies shows that many variants are relatively abundant among assumed healthy adults, and can therefore be excluded as pathogenic. A few variants were upgraded from VUSs to likely pathogenic variants. This is probably based on functional studies, discovery of the variants in more individuals with HBOC (or absence of the variants in healthy controls), and / or extensive segregation in families. For 15 of the variants from HUH there were no registered classifications in ClinVar. Most likely these variants are very rare and only occur in individuals/families in Western Norway. Since Norway has a relatively small population, there is often limited clinical information on a variant, while classification reports from the same variant in ClinVar can be based on a larger amount of information from several institutions and countries.

The *BRCA1* Norway collaboration has shown that data sharing increases the amount of evidence and contributes to national standardization and harmonization of variant classification and patient management. Data sharing is especially important for rare variants for which there is often limited evidence available. In small families, there are frequently insufficient family members to perform an informative segregation analysis. In addition, reduced penetrance for certain variants might add to the complexity. Consequently, such variants are often classified as VUSs. Hence, gathering of multiple observations of the same *BRCA1* variant and comparison of independent interpretations will increase the credibility of a given classification and increase the accuracy of cancer risk assessments. Data sharing can also help constrain laboratory resources.

To date, sharing information among the medical genetic departments in Norway has been limited, mostly due to the strict laws about the privacy of patients. When this project was initiated, the Norwegian law defined rare variants as information that may be used for the identification of individuals, and sharing of databases containing such information between different hospitals was therefore not allowed. Prior to initiating this study, the scientific community had though expressed a desire for increased sharing of data regarding variant interpretation between the laboratories, and currently there is a proposal to a change in the law that will make it possible to share such information. The major challenge is to find a common platform where important information about the variants can be exchanged in accordance with the guidelines for patient privacy policies in an efficient manner. The collaboration between all the diagnostic genetic laboratories in Norway will be extended to include variant interpretation of several other cancer genes like *BRCA2* and the *MMR* genes. At the clinical and diagnostic level, a national working group with participants from all departments of medical genetics in the field of hereditary cancer has already been established. In addition, a national network for hereditary cancer organised by the Norwegian Directorate of Health works on the national guidelines for different cancers to ensure that recommendations concerning genetics are up-to-date and communicated to non-genetic clinicians.

To summarize, the *BRCA1* Norway study shows that collaboration and data sharing can; (1) provide a more detailed overview of the *BRCA1* variant spectrum in the Norwegian population, (2) reveal discrepancies in variant interpretation among different laboratories, (3) reveal outdated classification reports and ensure up to date interpretations, (4) reduce the number of VUSs, (5) reduce time spent on variant interpretation, (6) ensure more trustworthy classifications in accordance with increasing information, and (7) guide patients and clinicians to make well-informed clinical decisions.

## Supplementary Information

Below is the link to the electronic supplementary material.

Supplementary file1 (DOCX 99 kb)

## Data Availability

The dataset generated and analysed during the current study is available from the corresponding author upon reasonable request.
