# Supplementary material for: BRCA1 Norway: comparison of classification for BRCA1 germline variants detected in families with suspected hereditary breast and ovarian cancer between different laboratories
Source: Fam Cancer. 2022 Jan 4;21(4):389–98. doi: 10.1007/s10689-021-00286-6 (PMC9636114; doi:10.1007/s10689-021-00286-6)
Supplement: Supplementary file 1 — Supplementary file1 (DOCX 99 kb) [file 10689_2021_286_MOESM1_ESM.docx]

**Supplementary table 1. Overview of all *BRCA1* variants found at the four genetic laboratories in Norway**

|  | **Oslo**  **(OUH)** | | **Bergen**  **(HUH)** | | **Tromsø**  **(UNN)** | | **Trondheim**  **(TUH)** | | **Reclassified** | |
| --- | --- | --- | --- | --- | --- | --- | --- | --- | --- | --- |
| **Variant** | **Class** | **Date** | **Class** | **Date** | **Class** | **Date** | **Class** | **Date** | **Class** | **Date** |
| c.-125C>T |  |  |  |  |  |  | 3 | 2016 |  |  |
| c.-107_-103del |  |  |  |  | 2 | 2012 |  |  |  |  |
| c.-86C>T |  |  |  |  |  |  | 3 | 2018 |  |  |
| c.-66C>G |  |  |  |  |  |  | 3 | 2018 |  |  |
| c.-20+11C>T |  |  |  |  |  |  | 3 | 2018 |  |  |
| c.-20+46G>C |  |  |  |  |  |  | 3 |  |  |  |
| c.-19-85_-19-81del |  |  | 2 | 2019 |  |  |  |  |  |  |
| c.-19-41T>C |  |  | 2 | 2017 |  |  |  |  |  |  |
| c.1A>G | 5 | 2018 | 5 | 2015 | 5 | 2009 |  |  |  |  |
| c.19C>T | 3 | 2015 | 2 | 2015 | 3 | 2018 |  |  | 3 | 2021 |
| c.65T>C | 5 | 2018 |  |  |  |  |  |  |  |  |
| c.66dup | 5 |  | 5 | 2018 |  |  | 5 | 2015 |  |  |
| c.68_69del | 5 | 2017 |  |  |  |  |  |  |  |  |
| c.69G>T | 3 | 2015 |  |  |  |  |  |  | 3 | 2019 |
| c.75C>T |  |  | 2 | 2018 | 2 | 2013 | 2 | 2017 |  |  |
| c.80+7C>A | 2 | 2017 |  |  |  |  |  |  |  |  |
| c.81-14C>T |  |  | 2 | 2018 | 2 | 2016 |  |  |  |  |
| c.81-13C>A | 2 | 2018 |  |  |  |  |  |  |  |  |
| c.81-12C>G | 2 | 2018 |  |  |  |  |  |  |  |  |
| c.81-11del | 2 | 2018 |  |  |  |  |  |  |  |  |
| c.81-2del |  |  | 5 | 2019 |  |  |  |  |  |  |
| c.114G>A |  |  | 2 | 2017 |  |  |  |  |  |  |
| c.115T>G | 4 | 2016 |  |  |  |  |  |  |  |  |
| c.116G>A | 5 | 2018 | 5 | 2015 |  |  |  |  |  |  |
| c.130T>A | 5 | 2018 |  |  |  |  | 5 | 2015 |  |  |
| c.133_134del |  |  | 5 | 2014 |  |  |  |  |  |  |
| c.140G>T | 4 | 2014 | 5 | 2008 |  |  |  |  | 4 | 2021 |
| c.147G>A |  |  | 3 | 2016 |  |  |  |  |  |  |
| c.154C>T |  |  |  |  |  |  | 3 | 2018 |  |  |
| c.169G>C |  |  |  |  | 3 | 2014 |  |  | 3 | 2019 |
| c.178C>T | 5 | 2017 |  |  |  |  |  |  |  |  |
| c.181T>G | 5 | 2019 | 5 | 2019 |  |  |  |  |  |  |
| c.199G>T |  |  | 3 | 2013 |  |  |  |  | 2 | 2019 |
| c.212+1G>T |  |  |  |  | 5 | 2018 |  |  |  |  |
| c.212+21G>A |  |  |  |  |  |  | 3 | 2018 |  |  |
| c.213-5T>A | 3 | 2019 |  |  |  |  |  |  |  |  |
| c.241C>T | 5 | 2012 |  |  |  |  |  |  |  |  |
| c.255G>A | 2 | 2019 |  |  |  |  |  |  |  |  |
| c.301+7G>A |  |  |  |  | 3 | 2013 | 2 | 2015 | 2^∆○^, 1^□^ | 2021 |
| c.302-124del |  |  |  |  | 2 | 2013 |  |  |  |  |
| c.302-2A>C | 4 | 2018 |  |  |  |  |  |  |  |  |
| c.305C>G | 3 | 2015 |  |  |  |  |  |  | 2 | 2019 |
| c.314A>G |  |  | 3 | 2014 |  |  |  |  | 2 | 2019 |
| c.334A>G | 2 | 2018 |  |  |  |  |  |  |  |  |
| c.386del |  |  | 5 | 2019 |  |  |  |  |  |  |
| c.397C>T |  |  | 3 | 2019 |  |  |  |  |  |  |
| c.441G>C | 3 | 2017 | 3 | 2013 | 2 | 2010 |  |  | 3 | 2021 |
| c.441+18C>T |  |  |  |  | 2 | 2013 |  |  |  |  |
| c.441+21C>T |  |  | 2 | 2018 |  |  | 3 | 2018 | 3 | 2021 |
| c.441+41dup |  |  | 5 |  |  |  |  |  |  |  |
| c.445G>T | 5 | 2015 | 5 | 2014 |  |  |  |  |  |  |
| c.448del | 5 | 2017 |  |  |  |  |  |  |  |  |
| c.457_458ins21 | 5 | 2014 | 3 | 2016 |  |  | 5 | 2016 | 5 | 2019 |
| c.457A>C |  |  | 3 | 2015 |  |  |  |  | 3 | 2019 |
| c.486G>T | 2 | 2017 |  |  |  |  |  |  |  |  |
| c.509G>A | 2 | 2017 |  |  |  |  |  |  |  |  |
| c.510del | 5 | 2016 |  |  |  |  | 5 | 2016 |  |  |
| c.514C>T | 5 |  |  |  |  |  | 5 | 2015 |  |  |
| c.538A>G | 3 | 2016 |  |  |  |  |  |  |  |  |
| c.547+2dup | 3 | 2017 |  |  |  |  |  |  |  |  |
| c.547+14del |  |  | 3 | 2011 | 2 |  |  |  | 2^∆^,1^□^ | 2021 |
| c.548-17G>T |  |  | 2 | 2016 | 2 | 2010 | 2 | 2017 |  |  |
| c.548-3del |  |  | 3 | 2016 |  |  |  |  |  |  |
| c.557C>A |  |  | 3 | 2014 |  |  | 2 | 2015 | 2 | 2021 |
| c.5586C>T | 2 | 2019 | 2 | 2019 |  |  |  |  |  |  |
| c.564A>G | 2 | 2015 |  |  |  |  |  |  |  |  |
| c.570C>T | 2 | 2014 | 2 | 2017 | 2 | 2011 |  |  |  |  |
| c.571G>A |  |  | 3 | 2012 |  |  |  |  | 2 | 2019 |
| c.591C>T |  |  |  |  | 2 | 2010 |  |  |  |  |
| c.594-34T>C |  |  | 2 | 2011 |  |  |  |  |  |  |
| c.594-20A>G | 2 | 2017 |  |  |  |  |  |  |  |  |
| c.670+7G>A | 2 | 2014 |  |  |  |  |  |  |  |  |
| c.670+16G>A |  |  | 3 | 2018 | 2 | 2010 | 2 | 2016 | 2 | 2021 |
| c.671-12del | 3 | 2015 |  |  |  |  |  |  |  |  |
| c.671-10A>G | 3 | 2019 |  |  |  |  |  |  |  |  |
| c.692C>T | 3 | 2015 |  |  |  |  |  |  | 3 | 2019 |
| c.697_698del | 5 | 2018 | 5 | 2018 | 5 | 2009 |  |  |  |  |
| c.712C>T | 3 | 2015 |  |  |  |  |  |  | 3 | 2019 |
| c.734A>T | 3 | 2014 |  |  |  |  |  |  | 3 | 2019 |
| c.736T>G |  |  | 3 | 2014 | 2 | 2010 | 2 | 2018 | 2 | 2021 |
| c.765G>A | 2 | 2018 |  |  |  |  |  |  |  |  |
| c.766A>T | 3 | 2018 | 3 | 2017 |  |  |  |  |  |  |
| c.794_795del | 5 | 2017 |  |  |  |  |  |  |  |  |
| c.814G>T |  |  | 5 | 2018 |  |  |  |  |  |  |
| c.825C>T | 2 | 2018 |  |  |  |  |  |  |  |  |
| c.834T>G | 2 | 2017 |  |  |  |  |  |  |  |  |
| c.843_846del | 5 | 2015 |  |  |  |  |  |  |  |  |
| c.848T>A | 5 |  |  |  |  |  |  |  |  |  |
| c.889A>G | 2 | 2019 | 3 | 2016 |  |  |  |  | 2*,3^∆^ | 2021 |
| c.914G>C |  |  | 3 | 2012 |  |  |  |  | 3 | 2019 |
| c.914G>T |  |  | 3 | 2012 |  |  |  |  | 3 | 2019 |
| c.929del |  |  | 5 | 2019 |  |  |  |  |  |  |
| c.995G>A | 2 | 2018 |  |  |  |  |  |  |  |  |
| c.1002del |  |  | 5 | 2016 |  |  |  |  |  |  |
| c.1016dup | 5 | 2018 | 5 | 2019 | 5 | 2018 | 5 | 2018 |  |  |
| c.1021G>A | 2 | 2018 |  |  |  |  |  |  |  |  |
| c.1040T>A | 3 | 2015 |  |  |  |  |  |  | 3 | 2019 |
| c.1040T>C |  |  |  |  |  |  | 3 | 2018 |  |  |
| c.1058G>A | 5 | 2018 |  |  |  |  |  |  |  |  |
| c.1059G>A | 5 | 2018 |  |  |  |  |  |  |  |  |
| c.1066C>T | 5 | 2017 |  |  |  |  |  |  |  |  |
| c.1072del | 5 | 2017 | 5 | 2015 |  |  |  |  |  |  |
| c.1076_1080del |  |  |  |  | 4 | 2014 |  |  |  |  |
| c.1081T>C | 2 | 2017 |  |  |  |  |  |  |  |  |
| c.1082_1092del | 5 | 2018 |  |  |  |  |  |  |  |  |
| c.1107_1111del |  |  |  |  | 4 | 2011 |  |  |  |  |
| c.1125A>G | 2 | 2015 |  |  |  |  |  |  |  |  |
| c.1149T>C | 2 | 2014 |  |  |  |  |  |  |  |  |
| c.1169A>C | 2 | 2018 |  |  |  |  |  |  |  |  |
| c.1175_1214del | 5 | 2015 |  |  |  |  |  |  |  |  |
| c.1196A>G | 3 | 2015 |  |  |  |  |  |  | 3 | 2019 |
| c.1242C>T | 2 | 2014 |  |  |  |  |  |  |  |  |
| c.1287del | 5 | 2008 | 5 | 2015 | 4 | 2014 |  |  | 5 | 2021 |
| c.1292dup | 5 | 2014 | 5 | 2019 |  |  |  |  |  |  |
| c.1333G>C |  |  |  |  |  |  | 3 | 2015 | 3 | 2019 |
| c.1360_1361del | 5 |  |  |  |  |  |  |  |  |  |
| c.1392C>T | 2 | 2016 |  |  |  |  |  |  |  |  |
| c.1405G>A | 3 | 2014 |  |  |  |  |  |  | 3 | 2019 |
| c.1419C>T | 2 | 2018 |  |  | 2 | 2015 | 2 | 2018 |  |  |
| c.1427A>G | 2 | 2018 |  |  |  |  |  |  |  |  |
| c.1434_1435del | 5 | 2015 |  |  |  |  |  |  |  |  |
| c.1441C>G | 2 | 2017 |  |  |  |  |  |  |  |  |
| c.1450G>T | 5 | 2017 | 5 | 2015 |  |  |  |  |  |  |
| c.1486C>T |  |  | 2 | 2019 |  |  |  |  |  |  |
| c.1487G>A |  |  | 2 | 2018 | 2 | 2009 | 2 | 2016 |  |  |
| c.1500T>A | 3 | 2013 |  |  |  |  |  |  | 3 | 2019 |
| c.1508A>G | 2 | 2017 |  |  |  |  | 3 | 2017 | 2*^○^,3^∆^ | 2021 |
| c.1510C>T | 3 | 2016 |  |  |  |  |  |  |  |  |
| c.1511G>A | 2 | 2018 |  |  |  |  |  |  |  |  |
| c.1521_1531del | 5 | 2017 |  |  |  |  |  |  |  |  |
| c.1533C>G |  |  | 2 | 2015 |  |  |  |  |  |  |
| c.1534C>T | 2 | 2018 | 2 | 2018 | 3 | 2015 | 3 | 2018 | 2 | 2021 |
| c.1556del | 5 | 2019 | 5 | 2018 | 5 | 2016 | 5 | 2018 |  |  |
| c.1567T>G | 3 | 2016 | 3 | 2014 |  |  |  |  |  |  |
| c.1568T>G | 2 | 2018 |  |  | 3 | 2017 | 3 | 2017 | 2*^○^,3^∆□^ | 2021 |
| c.1580A>G |  |  |  |  |  |  | 3 | 2015 | 3 | 2019 |
| c.1600C>T | 5 | 2016 |  |  |  |  |  |  |  |  |
| c.1616C>T | 2 | 2018 |  |  |  |  |  |  |  |  |
| c.1640A>C |  |  |  |  | 3 | 2019 |  |  |  |  |
| c.1674dup | 5 | 2012 |  |  |  |  |  |  |  |  |
| c.1687C>T | 5 | 2018 | 4 | 2013 | 5 | 2018 |  |  | 5 | 2021 |
| c.1695dup | 5 |  |  |  |  |  |  |  |  |  |
| c.1714G>A | 3 | 2016 |  |  |  |  |  |  |  |  |
| c.1722C>T |  |  |  |  |  |  | 3 | 2015 | 2 | 2019 |
| c.1723G>A |  |  | 3 | 2017 |  |  |  |  |  |  |
| c.1724A>G | 3 | 2017 |  |  |  |  |  |  |  |  |
| c.1745C>T | 3 | 2015 |  |  |  |  |  |  | 3 | 2019 |
| c.1746G>A | 2 | 2015 |  |  |  |  |  |  |  |  |
| c.1772T>C | 2 | 2019 | 3 | 2011 |  |  |  |  | 2 | 2021 |
| c.1793T>G | 5 | 2014 |  |  |  |  |  |  |  |  |
| c.1823_1826del | 5 | 2015 |  |  |  |  |  |  |  |  |
| c.1824_1826del | 3 | 2018 |  |  |  |  |  |  |  |  |
| c.1829G>C |  |  | 3 | 2014 |  |  |  |  | 3 | 2019 |
| c.1834A>G | 3 | 2016 |  |  |  |  |  |  |  |  |
| c.1840A>T |  |  |  |  |  |  | 5 | 2015 |  |  |
| c.1846_1848del | 2 | 2018 |  |  |  |  |  |  |  |  |
| c.1865C>T |  |  | 3 | 2015 |  |  |  |  | 3 | 2019 |
| c.1866G>A | 2 | 2018 |  |  | 2 | 2016 |  |  |  |  |
| c.1879G>A | 2 | 2018 | 3 | 2019 | 3 | 2016 |  |  | 2*,3^∆□^ | 2021 |
| c.188T>A | 5 | 2016 |  |  |  |  |  |  |  |  |
| c.1893A>C | 2 | 2016 |  |  |  |  |  |  |  |  |
| c.1901C>T |  |  | 3 | 2017 |  |  |  |  |  |  |
| c.1911T>C | 2 | 2018 | 2 | 2018 |  |  |  |  |  |  |
| c.1927A>G | 3 | 2015 |  |  |  |  |  |  | 3 | 2019 |
| c.1961del |  |  |  |  | 5 | 2011 |  |  |  |  |
| c.1961dup | 5 |  | 5 | 2009 |  |  |  |  |  |  |
| c.1978G>A | 3 | 2014 |  |  |  |  |  |  | 3 | 2019 |
| c.2006T>C |  |  | 3 | 2017 |  |  |  |  |  |  |
| c.2019del | 5 | 2018 |  |  |  |  |  |  |  |  |
| c.2024C>G | 3 | 2015 |  |  |  |  |  |  | 3 | 2019 |
| c.2027C>T | 3 | 2014 |  |  |  |  |  |  | 3 | 2019 |
| c.2043dup |  |  | 5 | 2015 |  |  |  |  |  |  |
| c.2050C>T | 3 | 2014 | 3 | 2013 | 3 | 2017 |  |  |  |  |
| c.2063C>A |  |  |  |  |  |  | 3 | 2018 |  |  |
| c.2083G>T | 2 | 2017 |  |  |  |  |  |  |  |  |
| c.2123C>A | 3 | 2015 |  |  |  |  | 3 | 2015 | 3 | 2019 |
| c.2123del |  |  | 5 | 2017 |  |  |  |  |  |  |
| c.2131A>C | 2 | 2019 | 3 | 2019 |  |  |  |  | 2*,3^∆^ | 2021 |
| c.2135G>A | 3 | 2015 |  |  | 3 | 2018 |  |  |  |  |
| c.2138C>G | 5 | 2016 |  |  |  |  |  |  |  |  |
| c.2140A>G |  |  |  |  | 3 | 2017 |  |  |  |  |
| c.2146A>G |  |  | 3 | 2008 |  |  |  |  | 3 | 2019 |
| c.2167A>G |  |  | 2 | 2012 |  |  | 2 | 2016 |  |  |
| c.2183G>A | 2 | 2018 |  |  | 3 | 2016 |  |  | 2*,3^∆□^ | 2021 |
| c.2185G>T | 5 | 2010 |  |  |  |  |  |  |  |  |
| c.2191_2227del |  |  | 5 | 2016 |  |  |  |  |  |  |
| c.2196del | 5 | 2018 |  |  |  |  |  |  |  |  |
| c.2245G>A | 3 | 2015 |  |  |  |  |  |  | 3 | 2019 |
| c.2252T>C | 2 | 2016 |  |  |  |  |  |  |  |  |
| c.2257dup | 5 | 2015 |  |  |  |  |  |  |  |  |
| c.2293G>T | 5 | 2015 |  |  |  |  |  |  |  |  |
| c.2312T>C | 2 | 2019 |  |  |  |  |  |  |  |  |
| c.2315T>C | 3 | 2015 |  |  |  |  | 2 | 2019 | 1*,2^∆○^ | 2021 |
| c.2336C>T |  |  |  |  |  |  | 3 | 2018 |  |  |
| c.2347A>G | 2 | 2016 |  |  | 2 | 2018 |  |  |  |  |
| c.2351_2357del | 5 | 2017 | 5 | 2019 |  |  |  |  |  |  |
| c.2352G>A | 2 | 2017 |  |  |  |  |  |  |  |  |
| c.2368A>G | 2 | 2016 |  |  |  |  |  |  |  |  |
| c.2389G>T | 5 |  |  |  |  |  |  |  |  |  |
| c.2403T>G | 3 | 2013 | 3 | 2014 |  |  | 3 | 2017 |  |  |
| c.2412G>C | 2 | 2016 |  |  |  |  |  |  |  |  |
| c.2428A>T |  |  | 2 | 2017 |  |  |  |  |  |  |
| c.2438dup | 5 |  | 5 | 2012 | 5 | 2014 | 5 | 2017 |  |  |
| c.2475del | 5 | 2017 | 5 | 2012 | 5 | 2016 |  |  |  |  |
| c.2477C>A |  |  | 2 | 2016 |  |  |  |  |  |  |
| c.2495C>T | 3 | 2010 |  |  |  |  |  |  | 3 | 2019 |
| c.2503C>T | 3 | 2014 |  |  |  |  |  |  | 3 | 2019 |
| c.2518A>G | 2 | 2016 |  |  |  |  |  |  |  |  |
| c.2521C>T |  |  | 2 | 2018 | 2 | 2009 | 2 | 2018 |  |  |
| c.2522G>A | 3 | 2015 |  |  |  |  |  |  | 2 | 2019 |
| c.2544A>C | 3 | 2011 |  |  |  |  |  |  | 3 | 2019 |
| c.2558ins356 | 5 | 2017 |  |  |  |  |  |  |  |  |
| c.2584A>G |  |  | 3 | 2013 |  |  |  |  | 2 | 2019 |
| c.2591C>G | 5 | 2018 |  |  |  |  |  |  |  |  |
| c.2606T>C | 3 | 2018 |  |  |  |  |  |  |  |  |
| c.2643del |  |  | 5 | 2010 |  |  |  |  |  |  |
| c.2662C>T |  |  | 3 | 2016 |  |  |  |  |  |  |
| c.2666C>T |  |  | 3 | 2018 |  |  |  |  |  |  |
| c.2668G>A |  |  | 3 | 2019 | 3 | 2017 |  |  |  |  |
| c.2681_2682del | 5 | 2015 | 5 | 2019 |  |  |  |  |  |  |
| c.2685_2686del | 5 | 2018 |  |  |  |  |  |  |  |  |
| c.2692_2693ins |  |  | 5 | 2017 |  |  |  |  |  |  |
| c.2692A>G |  |  |  |  |  |  | 3 | 2015 | 3 | 2019 |
| c.2727_2730del | 5 | 2017 |  |  |  |  |  |  |  |  |
| c.2765C>G |  |  | 3 | 2019 |  |  |  |  |  |  |
| c.2773A>G | 2 | 2017 | 3 | 2014 |  |  |  |  | 2*,3^∆^ | 2021 |
| c.2783G>A | 2 | 2017 |  |  |  |  |  |  |  |  |
| c.2798G>A | 2 | 2018 | 3 | 2016 |  |  |  |  | 2*,3^∆^ | 2021 |
| c.2814A>G | 2 | 2015 |  |  |  |  |  |  |  |  |
| c.2836A>G | 2 | 2016 |  |  |  |  |  |  |  |  |
| c.2849C>T | 2 | 2018 |  |  |  |  |  |  |  |  |
| c.2864C>A | 5 | 2015 |  |  |  |  |  |  |  |  |
| c.2869C>T | 5 | 2016 |  |  |  |  |  |  |  |  |
| c.2933dup |  |  | 5 | 2012 | 5 | 2018 |  |  |  |  |
| c.2981_2982del | 5 | 2015 |  |  |  |  |  |  |  |  |
| c.2989_2990dup | 5 | 2015 | 5 | 2009 |  |  | 5 | 2016 |  |  |
| c.3003A>G | 2 | 2014 | 2 | 2016 |  |  |  |  |  |  |
| c.3005del |  |  | 5 | 2015 |  |  |  |  |  |  |
| c.3022A>G |  |  | 2 | 2016 |  |  | 2 | 2017 |  |  |
| c.3041T>A | 3 | 2012 | 2 | 2016 |  |  |  |  | 3 | 2021 |
| c.3048_3052dup | 5 | 2018 | 5 | 2019 |  |  |  |  |  |  |
| c.3083G>A |  |  |  |  |  |  | 2 | 2016 |  |  |
| c.3084_3094del | 5 | 2018 | 5 | 2015 | 5 | 2009 | 5 | 2016 |  |  |
| c.3085A>G | 3 | 2016 |  |  |  |  |  |  |  |  |
| c.3119G>A |  |  |  |  | 2 | 2009 |  |  |  |  |
| c.3126C>G |  |  |  |  |  |  | 3 | 2018 |  |  |
| c.3178G>T | 5 | 2018 | 5 | 2015 |  |  | 5 | 2015 |  |  |
| c.3181del | 5 | 2018 |  |  |  |  |  |  |  |  |
| c.3185G>T | 3 | 2015 |  |  |  |  |  |  | 3 | 2019 |
| c.3210A>C |  |  | 2 | 2019 |  |  |  |  |  |  |
| c.3228_3229del | 5 | 2018 | 5 | 2019 | 4 | 2012 | 5 | 2018 | 5 | 2021 |
| c.3233C>T |  |  | 3 | 2012 |  |  |  |  | 3 | 2019 |
| c.3257T>A | 5 | 2019 |  |  |  |  |  |  |  |  |
| c.3270A>G | 2 | 2016 |  |  |  |  |  |  |  |  |
| c.3296C>T |  |  |  |  | 2 | 2013 |  |  |  |  |
| c.3302G>A |  |  | 2 | 2013 |  |  |  |  |  |  |
| c.3319G>T | 5 | 2018 |  |  | 4 | 2010 |  |  | 5 | 2021 |
| c.3327_3329del |  |  |  |  | 3 | 2013 |  |  |  |  |
| c.3327A>C | 2 | 2014 |  |  |  |  |  |  |  |  |
| c.3328_3330del | 2 | 2018 |  |  |  |  |  |  |  |  |
| c.3329dup | 5 | 2018 |  |  |  |  |  |  |  |  |
| c.3331_3334del | 5 | 2018 | 5 | 2015 | 5 | 2018 |  |  |  |  |
| c.3344_3346del | 3 | 2019 | 3 | 2018 |  |  |  |  |  |  |
| c.3377C>T | 3 | 2016 |  |  |  |  |  |  |  |  |
| c.3378A>G | 2 | 2016 |  |  |  |  |  |  |  |  |
| c.3392A>G |  |  |  |  |  |  | 3 | 2018 |  |  |
| c.3400G>T | 5 | 2015 |  |  |  |  |  |  |  |  |
| c.3407C>G |  |  | 3 | 2013 |  |  |  |  | 3 | 2019 |
| c.3418A>G |  |  | 2 | 2018 | 2 | 2009 | 2 | 2016 |  |  |
| c.3448C>T | 2 | 2018 |  |  |  |  |  |  |  |  |
| c.3454G>A | 2 | 2018 | 3 | 2018 |  |  |  |  | 2 | 2021 |
| c.3477_3479delAAAinsC | 5 | 2017 |  |  |  |  |  |  |  |  |
| c.3477A>C | 2 | 2016 |  |  |  |  |  |  |  |  |
| c.3541G>A | 3 | 2015 |  |  | 3 | 2019 |  |  |  |  |
| c.3544C>T | 5 | 2014 |  |  |  |  |  |  |  |  |
| c.3554A>G |  |  | 3 | 2019 |  |  |  |  |  |  |
| c.3555G>T | 2 | 2019 |  |  |  |  |  |  |  |  |
| c.3600G>C | 2 | 2018 |  |  |  |  |  |  |  |  |
| c.3607C>T | 5 | 2017 | 5 | 2018 | 5 | 2018 | 5 | 2016 |  |  |
| c.3608G>A | 3 | 2012 |  |  |  |  |  |  | 1 | 2019 |
| c.3629_3630del | 5 | 2015 |  |  |  |  |  |  |  |  |
| c.3640G>A | 3 | 2010 | 2 | 2019 |  |  |  |  | 2 | 2021 |
| c.3644_3648del | 5 | 2016 |  |  |  |  |  |  |  |  |
| c.3657G>C | 3 | 2012 |  |  |  |  |  |  | 2 | 2019 |
| c.3659A>T | 2 | 2018 |  |  | 3 | 2012 | 3 | 2018 | 2*,3^∆□○^ | 2021 |
| c.3689T>G | 5 |  |  |  |  |  |  |  |  |  |
| c.3700_3704del | 5 | 2017 |  |  |  |  | 5 | 2016 |  |  |
| c.3708T>G | 2 | 2018 |  |  | 2 | 2016 | 2 | 2015 |  |  |
| c.3710delT | 5 | 2016 |  |  |  |  |  |  |  |  |
| c.3713C>T |  |  | 3 | 2019 |  |  |  |  |  |  |
| c.3722C>A | 2 | 2019 |  |  |  |  |  |  |  |  |
| c.3740T>C | 2 | 2019 |  |  |  |  |  |  |  |  |
| c.3748G>A |  |  | 2 | 2017 | 2 | 2012 | 2 | 2019 |  |  |
| c.3756_3759del | 5 | 2015 | 5 | 2012 |  |  |  |  |  |  |
| c.3767C>T |  |  |  |  |  |  | 3 | 2019 |  |  |
| c.3770_3771del | 5 |  |  |  |  |  |  |  |  |  |
| c.3779del |  |  |  |  |  |  | 5 | 2015 |  |  |
| c.3813dup | 5 | 2015 |  |  |  |  |  |  |  |  |
| c.3817C>T | 5 |  |  |  |  |  |  |  |  |  |
| c.3824T>C |  |  |  |  |  |  | 3 | 2017 |  |  |
| c.3835del | 5 | 2017 |  |  |  |  |  |  |  |  |
| c.3874del | 5 |  |  |  |  |  |  |  |  |  |
| c.3889T>C | 3 | 2014 |  |  |  |  |  |  | 3 | 2019 |
| c.3937C>T |  |  | 5 | 2015 |  |  |  |  |  |  |
| c.3965A>G | 2 | 2018 |  |  |  |  |  |  |  |  |
| c.3966del | 5 | 2017 | 5 | 2018 |  |  |  |  |  |  |
| c.4035del | 5 | 2017 | 5 | 2014 | 5 | 2015 | 5 | 2017 |  |  |
| c.4036_4038del | 3 | 2017 |  |  |  |  |  |  |  |  |
| c.4036G>A | 3 | 2014 |  |  |  |  |  |  | 2 | 2019 |
| c.4039A>G |  |  | 2 | 2018 | 2 | 2009 |  |  |  |  |
| c.4045A>C | 3 | 2015 |  |  |  |  |  |  | 2 | 2019 |
| c.4065_4068del | 5 | 2019 | 5 | 2017 | 5 | 2017 | 5 | 2018 |  |  |
| c.4073A>G | 3 | 2018 |  |  |  |  |  |  |  |  |
| c.4096+3A>G | 3 | 2019 | 2 | 2017 |  |  | 3 | 2017 | 3 | 2021 |
| c.4096+18T>C |  |  | 2 | 2018 |  |  |  |  |  |  |
| c.4096+30C>T |  |  |  |  |  |  | 3 | 2018 |  |  |
| c.4097-20C>T | 2 | 2015 |  |  |  |  |  |  |  |  |
| c.4097-10G>A |  |  | 3 | 2014 |  |  |  |  |  |  |
| c.4097-2A>G |  |  |  |  | 5 | 2019 |  |  |  |  |
| c.4113G>A |  |  | 2 | 2014 |  |  | 2 | 2017 |  |  |
| c.4132G>A |  |  |  |  | 2 | 2013 |  |  |  |  |
| c.4146_4155dup | 5 | 2016 |  |  |  |  |  |  |  |  |
| c.4185+16G>A |  |  | 3 | 2019 |  |  |  |  |  |  |
| c.4185+21_4185+22del |  |  |  |  |  |  | 2 | 2018 |  |  |
| c.4185+30G>A |  |  | 3 | 2018 |  |  | 3 | 2018 |  |  |
| c.4186-11C>T |  |  |  |  | 2 | 2015 |  |  |  |  |
| c.4186C>T | 5 | 2014 |  |  |  |  |  |  |  |  |
| c.4288C>G | 2 | 2018 |  |  |  |  |  |  |  |  |
| c.4300del | 5 |  | 5 | 2015 | 4 | 2016 |  |  | 5 | 2021 |
| c.4308T>C |  |  |  |  | 2 | 2010 |  |  |  |  |
| c.4315C>T | 2 | 2019 | 3 | 2019 | 3 | 2018 | 3 | 2018 | 2*,3^∆□○^ | 2021 |
| c.4327C>G | 2 | 2014 |  |  |  |  |  |  |  |  |
| c.4327C>T | 5 | 2017 |  |  |  |  |  |  |  |  |
| c.4347A>G | 2 | 2014 |  |  |  |  |  |  |  |  |
| c.4357+17A>G |  |  | 2 | 2018 | 2 | 2015 | 2 | 2017 |  |  |
| c.4358-10C>T | 2 | 2017 |  |  |  |  |  |  |  |  |
| c.4364T>C | 2 | 2016 |  |  |  |  |  |  |  |  |
| c.4441G>A | 2 | 2016 |  |  |  |  |  |  |  |  |
| c.4484G>A | 5 | 2015 |  |  | 5 | 2015 | 4 | 2015 | 5 | 2021 |
| c.4484+3A>C | 4 | 2016 |  |  |  |  |  |  |  |  |
| c.4484+14A>G |  |  | 2 | 2018 |  |  |  |  |  |  |
| c.4484+61G>C |  |  | 3 | 2019 |  |  |  |  |  |  |
| c.4484+181_4484+182del |  |  |  |  | 2 | 2010 |  |  |  |  |
| c.4485-44C>T |  |  | 2 | 2014 |  |  |  |  |  |  |
| c.4485-10A>G | 3 | 2015 |  |  |  |  |  |  |  |  |
| c.4501T>A | 2 | 2018 |  |  |  |  |  |  |  |  |
| c.4508C>A | 5 | 2017 |  |  |  |  |  |  |  |  |
| c.4515T>C | 2 | 2016 |  |  |  |  |  |  |  |  |
| c.4532A>C | 2 | 2017 |  |  |  |  |  |  |  |  |
| c.4574_4575del |  |  | 5 | 2017 |  |  |  |  |  |  |
| c.4579G>T |  |  |  |  |  |  | 4 | 2015 |  |  |
| c.4603G>A | 3 | 2016 |  |  |  |  |  |  |  |  |
| c.4605G>A | 2 | 2018 |  |  |  |  |  |  |  |  |
| c.4612C>T |  |  | 5 | 2015 |  |  |  |  |  |  |
| c.4636G>A | 2 | 2016 |  |  |  |  |  |  |  |  |
| c.4636G>T | 2 | 2016 |  |  |  |  | 2 | 2018 |  |  |
| c.4644G>A | 2 | 2018 |  |  |  |  |  |  |  |  |
| c.4675+1G>A |  |  | 5 | 2017 |  |  |  |  |  |  |
| c.4675+28A>G |  |  | 2 | 2013 |  |  |  |  |  |  |
| c.4675G>A | 5 | 2018 |  |  |  |  |  |  |  |  |
| c.4676-19C>T | 2 | 2014 |  |  |  |  |  |  |  |  |
| c.4676-8C>G | 2 | 2016 |  |  |  |  |  |  |  |  |
| c.4676-7C>T | 2 | 2015 |  |  |  |  |  |  |  |  |
| c.4683C>A |  |  |  |  |  |  | 2 | 2016 |  |  |
| c.4689C>G | 5 |  | 5 | 2015 |  |  |  |  |  |  |
| c.4718A>G | 2 | 2018 |  |  |  |  |  |  |  |  |
| c.4725T>G |  |  | 2 | 2015 |  |  |  |  |  |  |
| c.4745del | 5 | 2019 | 5 | 2019 | 5 | 2010 |  |  |  |  |
| c.4750G>T | 3 | 2011 |  |  |  |  |  |  | 2 | 2019 |
| c.4765C>T | 2 | 2018 |  |  |  |  |  |  |  |  |
| c.4766G>A | 2 | 2017 |  |  |  |  |  |  |  |  |
| c.4775del |  |  | 5 | 2019 |  |  |  |  |  |  |
| c.4798T>C | 2 | 2016 |  |  |  |  |  |  |  |  |
| c.4799T>A |  |  | 4 | 2014 |  |  | 4 | 2015 |  |  |
| c.4812A>G |  |  | 2 | 2013 | 2 | 2010 |  |  |  |  |
| c.4837A>G |  |  |  |  | 2 | 2010 |  |  |  |  |
| c.4860T>C | 2 | 2016 |  |  |  |  |  |  |  |  |
| c.4882A>G | 2 | 2016 |  |  |  |  |  |  |  |  |
| c.4883T>C |  |  | 2 | 2018 | 2 | 2013 |  |  |  |  |
| c.4884G>A |  |  | 3 | 2017 |  |  |  |  |  |  |
| c.4930G>T |  |  |  |  |  |  | 5 | 2017 |  |  |
| c.4932_4933dup | 5 | 2013 |  |  |  |  |  |  |  |  |
| c.4941C>A |  |  |  |  | 2 | 2013 |  |  |  |  |
| c.4956G>A |  |  |  |  | 2 | 2009 |  |  |  |  |
| c.4964_4982del |  |  | 5 | 2019 |  |  |  |  |  |  |
| c.4964C>T | 4 | 2016 |  |  |  |  |  |  |  |  |
| c.4972del | 5 | 2019 | 5 | 2007 |  |  |  |  |  |  |
| c.4986+1G>T | 5 | 2014 | 5 | 2015 |  |  |  |  |  |  |
| c.4987-20A>G |  |  | 2 | 2018 |  |  |  |  |  |  |
| c.4987-4T>G | 2 | 2018 |  |  |  |  |  |  |  |  |
| c.4992C>T |  |  |  |  | 2 | 2016 |  |  |  |  |
| c.5002T>C |  |  |  |  | 3 | 2017 |  |  |  |  |
| c.5005G>T | 2 | 2018 |  |  |  |  |  |  |  |  |
| c.5017_5019del | 4 | 2018 |  |  |  |  |  |  |  |  |
| c.5030_5033del | 5 | 2017 |  |  |  |  |  |  |  |  |
| c.5030_5033dup | 5 | 2017 |  |  |  |  |  |  |  |  |
| c.5037A>G |  |  |  |  |  |  | 2 | 2017 |  |  |
| c.5047G>T | 5 | 2018 | 5 | 2008 | 4 | 2014 |  |  | 5 | 2021 |
| c.5049G>A | 2 | 2017 |  |  |  |  |  |  |  |  |
| c.5074+2T>C | 4 | 2014 |  |  |  |  |  |  |  |  |
| c.5075-53C>T |  |  | 2 | 2019 |  |  |  |  |  |  |
| c.5075-2A>C | 5 | 2018 | 5 | 2015 | 5 | 2014 | 5 | 2017 |  |  |
| c.5075A>C | 4 | 2017 |  |  |  |  |  |  |  |  |
| c.5095C>T | 5 | 2017 | 5 | 2014 |  |  |  |  |  |  |
| c.5096G>A | 4 | 2018 | 4 | 2013 | 5 | 2019 | 5 | 2017 | 5 | 2021 |
| c.5100A>G | 2 | 2019 |  |  |  |  |  |  |  |  |
| c.5117G>A |  |  | 4 | 2016 |  |  |  |  |  |  |
| c.5117G>C | 2 | 2017 |  |  | 2 | 2015 |  |  |  |  |
| c.5123C>A |  |  | 4 | 2010 |  |  |  |  |  |  |
| c.5123C>T | 3 | 2018 |  |  |  |  |  |  |  |  |
| c.5124G>A | 2 | 2015 |  |  |  |  |  |  |  |  |
| c.5125G>A | 3 | 2017 | 3 | 2016 | 3 | 2015 |  |  |  |  |
| c.5131A>C | 3 | 2017 |  |  |  |  |  |  |  |  |
| c.5153-31A>G |  |  | 3 | 2012 |  |  | 3 | 2017 |  |  |
| c.5153-26A>G |  |  | 3 | 2018 |  |  |  |  |  |  |
| c.5153-1G>C |  |  | 5 | 2019 |  |  |  |  |  |  |
| c.5153G>C | 4 | 2015 | 4 | 2008 |  |  |  |  |  |  |
| c.5158A>G |  |  |  |  | 2 | 2018 |  |  |  |  |
| c.5175A>G | 2 | 2016 | 2 | 2018 |  |  |  |  |  |  |
| c.5193+2del | 5 | 2016 |  |  |  |  |  |  |  |  |
| c.5193+43_5193+46del |  |  | 3 | 2019 |  |  |  |  |  |  |
| c.5194-30T>C |  |  |  |  |  |  | 3 | 2016 |  |  |
| c.5212G>A | 4 | 2017 |  |  |  |  |  |  |  |  |
| c.5213G>A | 5 |  | 4 | 2010 |  |  |  |  | 5 | 2021 |
| c.5248A>T |  |  |  |  | 4 | 2018 |  |  |  |  |
| c.5251C>T | 5 | 2018 |  |  |  |  |  |  |  |  |
| c.5258G>C | 4 | 2018 |  |  |  |  | 4 | 2017 |  |  |
| c.5266dup | 5 | 2018 | 5 | 2019 | 5 | 2018 | 5 | 2018 |  |  |
| c.5277+5A>G |  |  | 2 | 2014 |  |  |  |  |  |  |
| c.5277+48_5277+59dup |  |  |  |  | 2 | 2013 |  |  |  |  |
| c.5277+48_5277+60dup |  |  | 2 | 2012 |  |  |  |  |  |  |
| c.5278-14C>G | 2 | 2018 | 2 | 2014 |  |  | 2 | 2018 |  |  |
| c.5306A>G | 2 | 2017 |  |  |  |  |  |  |  |  |
| c.5309G>T | 4 | 2018 |  |  |  |  |  |  |  |  |
| c.5326C>T | 2 | 2018 |  |  |  |  |  |  |  |  |
| c.5332+4A>G | 3 | 2017 |  |  |  |  | 3 | 2019 |  |  |
| c.5333-8C>T | 2 | 2015 |  |  |  |  |  |  |  |  |
| c.5333-3del |  |  |  |  |  |  | 3 | 2018 |  |  |
| c.5346G>A | 5 | 2018 |  |  |  |  |  |  |  |  |
| c.5347A>C | 2 | 2017 |  |  |  |  |  |  |  |  |
| c.5348T>C | 2 | 2017 | 3 | 2018 |  |  |  |  | 2 | 2021 |
| c.5377A>T | 5 | 2017 |  |  |  |  |  |  |  |  |
| c.5406+8T>C |  |  | 2 | 2014 |  |  |  |  |  |  |
| c.5406+33A>T |  |  | 2 | 2018 |  |  |  |  |  |  |
| c.5407-36G>T |  |  | 2 | 2018 |  |  |  |  |  |  |
| c.5407-25T>A | 4 | 2018 | 4 | 2019 |  |  |  |  |  |  |
| c.5407-10G>A | 5 | 2017 |  |  |  |  |  |  |  |  |
| c.5407-2A>G | 5 | 2015 |  |  |  |  | 5 | 2016 |  |  |
| c.5411T>A |  |  |  |  | 2 | 2018 |  |  |  |  |
| c.5412C>T | 2 | 2017 | 2 | 2019 |  |  |  |  |  |  |
| c.5429T>C | 3 | 2018 |  |  |  |  |  |  |  |  |
| c.5434C>G | 5 | 2018 |  |  |  |  |  |  |  |  |
| c.5467+148del |  |  |  |  | 2 | 2012 |  |  |  |  |
| c.5477A>T | 2 | 2017 |  |  | 3 | 2016 | 3 | 2017 | 2 | 2021 |
| c.5503C>T | 5 | 2014 | 5 | 2019 |  |  |  |  |  |  |
| c.5504G>A | 3 | 2018 | 3 | 2018 | 3 | 2017 |  |  |  |  |
| c.5511G>A | 5 | 2019 | 5 | 2019 |  |  |  |  |  |  |
| c.5511G>T |  |  | 4 | 2014 |  |  |  |  |  |  |
| c.5513T>G | 4 | 2017 | 4 | 2017 |  |  | 4 | 2017 |  |  |
| c.5534del | 5 | 2018 | 5 | 2019 |  |  |  |  |  |  |
| c.5535C>A |  |  | 5 | 2015 |  |  |  |  |  |  |
| c.5576C>G |  |  | 3 | 2019 |  |  | 3 | 2018 |  |  |
|  |  |  |  |  |  |  |  |  |  |  |

**Note:** For *BRCA1* variants with conflicting classifications after reassessment, the following symbols indicate the corresponding laboratories: * = OUH, ^∆^ = HUH, ^□^ = UNN, ^○^ = TUH.
